# Dental Literature of the Day

**Published:** 1873-09

**Authors:** W. H. Jackson

**Affiliations:** Ann Arbor, Mich.


### DENTAL LITERATURE OF THE DAY.

BY W. H. JACKSON, ANN ARBOR, MICH.

In looking over the different Dental Journals which come to my hands, there seems to be a general want of precise conclusion, practical results and evidence of accurate observations; in too many of the articles, whether contributions or editorials, there is a lack of arriving at something excellent that the reader can appropriate for future use.

There is also a tendency to brag about the rapid strides the dental profession is making. Of what benefit is it to this or that one to read several pages in nearly every journal on the great advance of the dentistry of to-day, over that of a few years since. I will admit that an occasional article of this kind is a good stimulant to arouse readers to do their share in the great work of investigation; but in excess, it acts like that other stimulant, alcohol, which first exhilirates, then sickens, and is followed by a sedative effect, and all who drink at its fountain think that they know all, and there is no use of going any farther. The next month they get another draught of the same elixir, and so on the year round ; so that from nearly every one's mouth, comes forth those ominous words *our glorious* profession.

This continual singing of the near approach toward perfection, will soon leave us in the same dilemma as the cricket in the old story. After singing away all of the summer, the chilling winds of autumn came, and found him without the winter's store laid by. He called upon the neighboring ants for relief, but received in reply ; "We ants never borrow, and we ants never lend."

The profession will be unworthy its name, unless there is a little more earnest work and investigation.

There is a habit among writers of spreading over three or four pages of the journal, that which ought to have been contained on the half page; there is too much subject, and too little object ; or in other words, too much baseless theory, and too little practice and practical investigation. I have read today an article on a subject, which has puzzled me very much—one which I have sought in vain to understand. The writer covers three or four pages with the *subjective*, but when it comes to the *objective*, he sums it up after this manner: "Harris says, 'But we know of no treatment that will control or arrest this singular disease etc.,' but with our present knowledge of the disease, we can usually at least arrest its progress." Now this writer knows how to arrest (at least he so claims,) that which it is my greatest desire to learn to do, yet he does not make me any the wiser, as he gives no treatment or remedy to remove the disease.

The object of dental literature should be to teach those who study it, thereby elevating the profession generally. Those who profess to be teachers of a science, should treat the subject with which they are dealing. in a scientific manner, and not write for the sake of writing or filling so much space in a journal with nicely worded language, containing nothing to feed the mind or benefit the body of the reader or patient. What do I learn from an article on alveolar abscess, if the writer neglects to give his mode of treatment. What would you learn were I to tell you it is easy enough to arrest a case of spontaineous abrasion, with the present knowledge we have of the disease, if I do not give the treatment employed? Yet such is the manner in which many of the contributors of our dental journals write. Of course they expect that we will drink it down and say that it is all "O. K." because the learned so and so says so.

The more concise the scientific writer, the greater the value of his papers, for there is less slag and drop in proportion to the pure metal; as some ores are worthless on account of the great amount of impurities which they contain, so some authors are worthless on account of being too voluminous. Those writers who stand highest among scientific men, are those who are most concise and exact, for the scientist has to deal with facts and laws, and the fewer the words which will express these facts and laws, the greater their value; for if one instrument will perform the work of a dozen, any one would be foolish if he did not pass by the dozen and take up the one.

It should be our great aim to make our dental journals overflowing fountains of the latest professional information, to which all of us lesser lights may come for instruction and stimulation.

